# Synchronous Double Primary Angiosarcoma Originating from the Stomach and Rectum: A Case Report and a Literature Review

**DOI:** 10.34172/aim.2024.25

**Published:** 2024-03-01

**Authors:** Tanju Kapagan, Nilufer Bulut, Gokmen Umut Erdem, Suleyman Yıldırım, Zeynep Betul Erdem, Halil Sahin

**Affiliations:** ^1^Başakşehir Çam and Sakura City Hospital, Department of Medical Oncology, 34480 Istanbul, Turkey; ^2^Başakşehir Çam and Sakura City Hospital, Department of Gastroenterology, 34480 Istanbul, Turkey; ^3^Başakşehir Çam and Sakura City Hospital, Department of Pathology, 34480 Istanbul, Turkey

**Keywords:** Cancer, Gastrointestinal bleeding, Sarcoma, Synchronous angiosarcoma

## Abstract

Angiosarcomas originating from the gastrointestinal tract are rare but highly aggressive tumors with poor prognosis. These tumors can be misdiagnosed as benign and malignant gastrointestinal tract lesions. The definitive histological diagnosis of angiosarcomasis made by pathologists based on immunohistochemical analysis demonstrating cluster of differentiation 31 (CD31), factor VIII-related antigen (FVIIIRAg), erythroblast transformation specific related gene (ERG), and cluster of differentiation 34 (CD34). Angiosarcomas are treated with a single or multimodality approach that may include resection, radiotherapy, chemotherapy, and palliative care, depending on the stage of disease and the condition of the patient. No matter the treatment option, metastasis and death rates are substantially highin patients with angiosarcoma. In this context, a 59-year-old male with synchronous double primary angiosarcoma arising from the gastric and rectum who presented with the complaint of abdominal pain and distention to the outpatient clinic is presented in this case report, along with a brief literature review.

## Introduction

 Angiosarcomas are aggressive tumors arising from blood and lymphatic vessels,^1,2^ often observed in men in the sixth and seventh decades.^3-5^ These tumors are likely to be confused with ulcerous lesions since they are rarely encountered in daily clinical practice, and thus cancer diagnosis is often missed.^6^ Angiosarcomas may be treated with a single or multi-modality approach that primarily involves chemotherapy, including anthracyclines, dacarbazine, cisplatin, vinca alkaloids, thalidomide agents, or tyrosine kinase inhibitors such as pazopanib, sorafenib, or sunitinib, along with surgical resection, radiotherapy, plasma argon coagulation, and palliative care.^7,8^ In light of the foregoing information, a 59-year-old male diagnosed with angiosarcoma both in the stomach and rectum with treatment unresponsiveness and short survival has been addressed in this case report.

## Case Report

**Figure 1:**
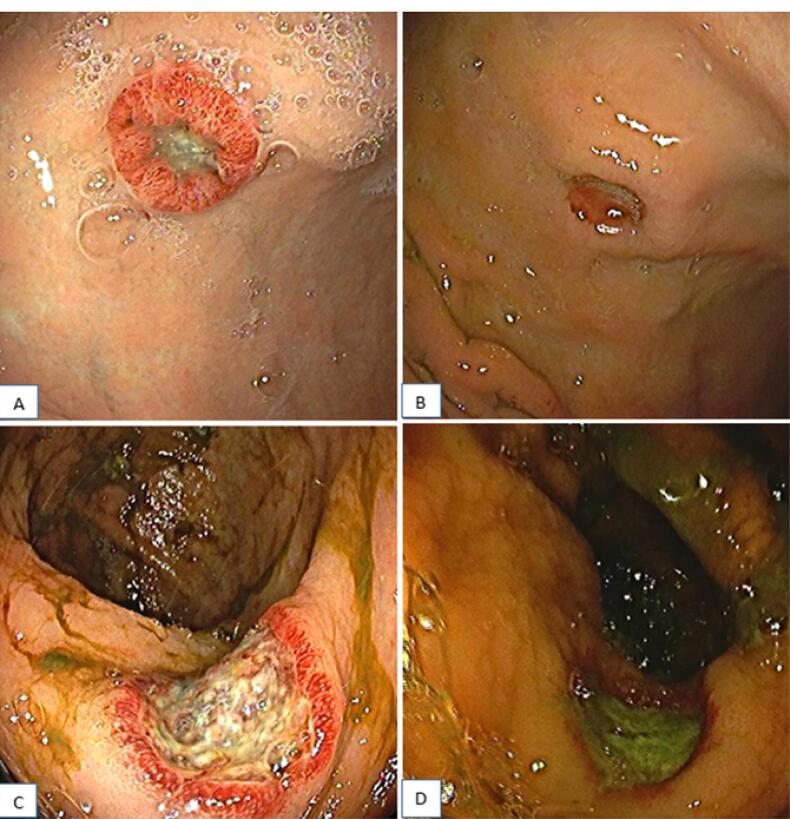
The Endoscopic Findings of Angiosarcoma in the Stomach (A, B) and Rectum (C, D) Before (A, C) and After (B, D) Treatment

 A 59-year-old male patient presented with a complaint of abdominal pain and distension. Abdominal ultrasonography revealed multiple cystic lesions in the hepatic and splenic parenchyma and free fluid in the abdomen. Follow-up magnetic resonance imaging demonstrated a massive amount of free fluid in the abdomen and multiple cystic lesions in the hepatic and splenic parenchyma, the largest of which measured 60 × 51 mm. The signal intensity of the lesions was hypointense on the T1-weighted image and hyperintense on the T2-weighted image. In addition, an increase in the symmetrical wall thickness was detected in the distal rectum. Upper endoscopy and colonoscopy examinations were requested for gastrointestinal (GI) tract cancer screening. Gastroscopy revealed a 1.5-cm ulcerated lesion that was surrounded by a raised hyperemic halo in the distal stomach corpus (Figure 1A), and colonoscopy represented a diminutive polyp in the rectum (Figure 1B).

**Figure 2:**
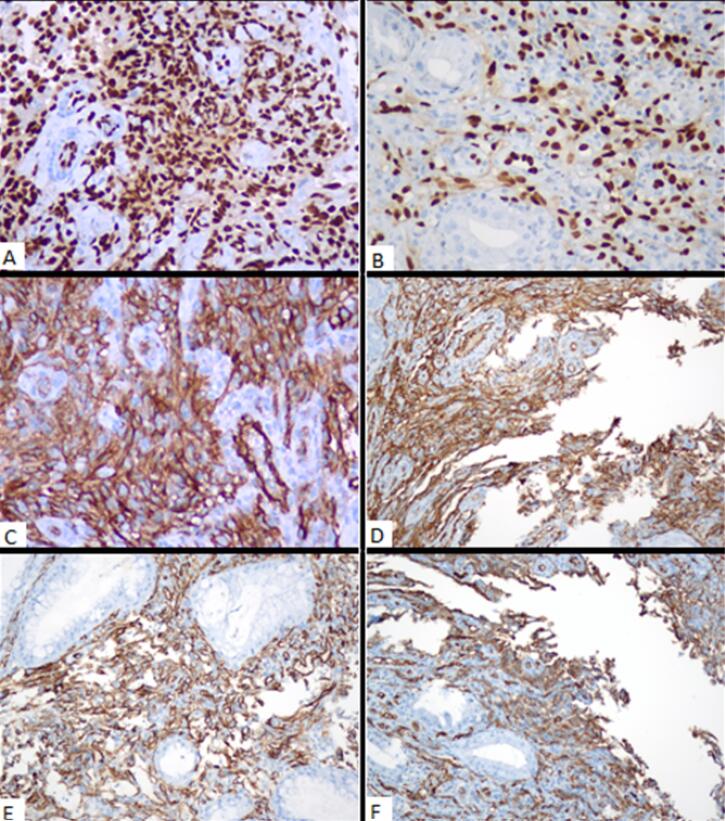
ERG (A, B), CD31 (C, D), and CD34 (E, F) Immunohistochemical Findings of Angiosarcoma in the Stomach and Rectum, Respectively

 Biopsy samples taken from the stomach and rectum had similar pathological features, suggesting endothelial angiosarcoma. Histopathologic examination of both localizations (i.e., stomach and rectum) revealed focally ulcerated and hyperplastic epithelium with a tumoral infiltration in the lamina propria. Neoplastic cells were spindled to be epithelioid-shaped with large, hyperchromatic, and pleomorphic nuclei, forming solid sheets, focally cleft-like spaces, and rarely intracytoplasmic lumens, expanding lamina propria around gastric and rectal crypts and glands. On immunohistochemical analyses, both tumors showed diffuse ERG (Figure 2 A-B), CD31 (Figure 2 C-D), and CD34 (Figure 2 E-F) expression, whereas cytokeratin [anti-cytokeratin monoclonal antibodies (AE1/AE3)], epithelial membrane antigen (EMA), human herpesvirus 8 (HHV8), special AT (adenine and thymine)-rich sequence-binding protein 2, sex-determining region Y-related high-mobility group box 10 protein (SOX10), 100% soluble protein (S100), and cytomegalovirus were all negative. Cytologic analysis of abdominal fluid and the fine needle aspiration biopsy taken from the cystic lesion demonstrated hemorrhagic effusion and mixed-type inflammatory cells in the hepatic region, respectively. The patient was started on 75 mg/m^2^ of doxorubicin once every 3 weeks. However, control gastroscopy and colonoscopy did not reveal any significant improvement in the gastric and rectal lesions after the completion of three cycles of treatment (Figure 1C-D). On the other hand, the positron emission tomography and computed tomography scans performed after the treatment revealed an increase in the 18F-fluorodeoxyglucose uptake in newly developed metastatic nodes in the supra/infra diaphragmatic regions. The patient’s general condition gradually deteriorated, and he died four months after diagnosis.

## Literature Review and Discussion

 A thorough review of the literature revealed 23 cases diagnosed with synchronous double primary angiosarcoma originating from the GI system (Table 1).^8-28^

**Table 1:** Cases of Synchronous Double Primary Angiosarcoma of the Gastrointestinal Tract (Modified From Zacarias Fohrding L et al^19^)

**Reference**	**Age (Years)/Gender**	**Initial Symptoms**	**Tumor Localizations**	**IHC**	**Radiation History**	**Treatment**	**Outcome**
Fukita et al^9^	80/M	Melena and dyspnea	Gastric, duodenum, and large bowel	FVIIIRAg, CD31, and CD34	None	None (PC)	Died shortly
De Francesco et al^10^	68/M	Haemoptysis and melena	Gastric, small bowel, lung, and thyroid	CD31	None	Chemotherapy	Died after 75 days
Navarro-Chagoya et al^11^	45/M	Melena and weight loss	Small bowel (multifocal)	FVIIIRAg, CD31, and pancytokeratin	Radiation	Resection	NA
Leong et al^12^	60/M	Dyspnea and weakness	Gastric and duodenum	CD31, vimentin, and pancytokeratin	NA	Resection	NA
Taxy and Battifora ^13^	64/M	GI bleeding	Small bowel (multifocal)	FVIIIRAg and vimentin	None	Resection	Died after one year
Taxy and Battifora ^13^	57/M	NA	Ileocecal valve, small bowel, and mesentery	FVIIIRAg	None	Resection	Died in several days
Louie et al^14^	78/M	Failure to thrive	Large bowel (multifocal)	ERG, CD31	None	None (PC)	Died shortly
Brown et al^15^	77/M	Rectal bleeding and obstructive bowel	Rectum and sigmoid colon	NA	NA	Resection	Died after 6 months
Chen et al^16^	77/F	Melena and dizziness	Gastric and large bowel	Pancytokeratin, CD31, CD34, EMA, and vimentin	None	Refused chemotherapy (PC)	Died after 3 months
Radić et al^17^	61/M	Fever (39°C), sweats, and vomiting	Large bowel (multifocal)	ERG, CD31, CD34, vimentin, and CD117	None	Resection	Died after 60 days
Pagni et al^18^	74/M	Anemia and melena	Gastric and recuml	FVIIIRAg and CD31	NA	None (PC)	NA
Zacarias Föhrding et al^19^	84/M	Anemia and melena	Small bowel (multifocal)	CD31, vimentin, and CD34	None	Resection	NA
Martínez-Alcalá García et al^20^	78/M	Anemia	Duodenum and jejunum	CD31	None	None (PC)	Died after 4 months
Wolov et al^21^	80/F	Peripheral edema and abdominal distension	Small and large bowel	FVIIIRAg	Radiation	Resection	Died after 2 weeks
Wolov et al^21^	69/F	Anorexia, weight loss, and hematochezia	Small and large bowel	FVIIIRAg	Radiation	Resection	Died after 23 days
Shi et al^22^	62/M	Appetite, weakness, melaena, and weight loss	Jejunum (multifocal)	Vimentin, CD31, CD34, and pancytokeratin	None	Resection	NA
Policarpio-Nicolas et al^23^	51/F	Appetite and abdominal pain	Small bowel and appendix	FVIIIRAg, CD31, and CD34	Radiation	Resection	Died after 10 months
Takahashi et al^24^	85/M	Fever (40°C) and abdominal distension	Small bowel (multifocal)	FVIIIRAg, CD31, CD34, and vimentin	None	Resection	Died after 42 days
Mohammed et al^25^	25/F	Abdominal pain and weight loss	Small and large bowel	NA	None	Resection	Died after 11 days
Delvaux et al^26^	67/M	Weight loss, abdominal pain, and melena	Small bowel (multifocal)	FVIIIRAg, CD31, and CD34	None	Resection	Died after 3 months
Al Ali et al^27^	87/M	Weakness	Duodenum and jejunum	NA	None	Endoscopy, argon plasma coagulation	Died after 6 weeks
Grewal et al^28^	73/M	Weakness and melena	Duodenum and jejunum	NA	Radiation	Resection	Died after 4 months
Nai et al^8^	73/M	Chest pain, dyspnea, and melena	Duodenum and jejunum	CD34, vimentin, Wilm’s tumor-1, and Vwf	None	Resection	Died in a short time
Present case	59/M	Abdominal distention and pain	Gastric and rectum	ERG, CD31, and CD34	None	Chemotherapy	Died after 4 months

*Note*. EMA: Epithelial membrane antigen; ERG: Erythroblast transformation specific related gene; FVIIIRAg: Factor VIII-related antigen; IHC: Immunohistochemical; NA: Not available; M: Male; F: Female; PC: Palliative care; Vwf: von Willebrand factor.

 Demographic characteristics, initial symptoms, and immunohistochemical features of our case and these 23 cases, as well as the applied treatment approaches, were tabulated using descriptive statistics (Table 2).

**Table 2:** Patients’ Age, Gender, Symptoms, Treatments, and Immunohistochemical Features

	**Age**		
	**Mean (y)**	**Median (y)**	**Minimum (y)-Maximum (y)**
Gender			
Female (n = 5)	60	69	25-80
Male (n = 19)	70	73	45-87
		**N**	**Percent**
Symptoms			
GI bleeding		13	54.20
Weight loss		5	20.80
Weakness		4	16.70
Abdominal pain		4	16.70
Anemia		3	12,.50
Dyspnea		3	12.50
Abdominal distansiyon		3	12.50
Appetite		2	8.30
Fever		2	8.30
Anorexia		1	4.20
Chest pain		1	4.20
Dizziness		1	4.20
Failure to thrive		1	4.20
Obstructive bowel		1	4.20
Peripheral edema		1	4.20
Sweats vomiting		1	4.20
IHC markers			
CD31		15	62.50
FVIIIRAg		10	41.60
CD34		10	41.60
Vimentin		8	33.30
Pancytokeratin		4	16.70
ERG		3	12.50
CD117		1	4.20
CKAE1/AE3		1	4.20
EMA		1	4.20
Vwf		1	4.20
Wilm’s tumor-1		1	4.20
NA		4	16.70
Treatments			
Resection		16	66.7
Palliative care		5	20.8
Chemotherapy		2	8.3
Endoscopic argon plasma coagulation		1	4.2

*Note*. EMA: Epithelial membrane antigen; ERG: Erythroblast transformation specific related gene; FVIIIRAg: Factor VIII-related antigen; GI: Gastrointestinal; HC: Immunohistochemical; NA: Not available; M: Male; F: Female; vWF: von Willebrand factor.

 In a study conducted by Schizas et al with 110 patients with angiosarcoma originating from the GI tract, of whom nearly 60% were male, whose mean age was approximately 62 years. In addition, 14 patients were diagnosed with synchronous double primary angiosarcoma originating from the GI system. The 6-month survival rate of these 14 patients, who mainly had symptoms such as GI bleeding, abdominal pain, and obstructive symptoms, was 23.08%. The univariate and multivariate logistic regression analyses revealed that surgical tumor resection was a significant factor in patients’ survival rates.^29^

 In another study conducted on 25 angiosarcoma patients, a strong correlation was found between the presence of angiosarcoma and CD31 and ERG among the immunohistochemical parameters.^30^

 In a systematic review, including 33 patients with primary colorectal angiosarcoma, it was concluded that the best treatment modality is either stand-alone complete surgical resection or complete surgical resection in combination with post-surgical chemotherapy.^31^

 Another systematic review, including 12 patients with primary small bowel angiosarcoma, reported that 9 of the 10 patients who were treated with only one of the following treatment modalities of surgery, chemotherapy, or argon plasma coagulation died within 1 year.^24^

## Conclusion

 In conclusion, combination treatment with surgical resection remains a cornerstone treatment strategy for angiosarcomas. However, it is important to note that the rate of local recurrence, metastasis, or death as a result of diagnostic delays due to the aggressive nature of tumors is still extremely high regardless of the treatment options used alongside surgical resection.
